# Surface Reflectance: An Optical Method for Multiscale Curvature Characterization of Wear on Ceramic–Metal Composites

**DOI:** 10.3390/ma13051024

**Published:** 2020-02-25

**Authors:** Julie Lemesle, Frederic Robache, Gaetan Le Goic, Alamin Mansouri, Christopher A. Brown, Maxence Bigerelle

**Affiliations:** 1 CNRS UMR 8201—LAMIH—Laboratoire d’Automatique, de Mécanique et d’Informatique Industrielles et Humaines, Université Polytechnique Hauts-de-France, F–59313 Valenciennes, France; Julie.Lemesle@uphf.fr (J.L.); Frederic.Robache@uphf.fr (F.R.); 2 EA 7535—ImViA—Laboratoire Imagerie et Vision Artificielle, Université de Bourgogne, 21078 Dijon CEDEX, France; Gaetan.le-Goic@ubfc.fr (G.L.G.); alamin.mansouri@u-bourgogne.fr (A.M.); 3 Surface Metrology Laboratory, Worcester Polytechnic Institute, Worcester, MA 01609, USA; brown@wpi.edu

**Keywords:** roughness, metal matrix composite, wear, reflectance transformation imaging, peak curvature

## Abstract

Surface gradient characterization by light reflectance (SGCLR) is used for the first time for multiscale curvature calculations and discrimination of worn surfaces on six damaged ceramic–metal composites. Measurements are made using reflectance transformation imaging (RTI). Slope and curvature maps, generated from RTI, are analyzed instead of heights. From multiscale decompositions, bootstrapping, and analysis of variance (ANOVA), a strong correlation (*R²* = 0.90) is found between the density of furrows of Mehlum curvatures, with a band pass filter at 5.4 µm, present in ceramic grains and their mechanical properties. A strong correlation is found between the mean curvatures of the metal and the ceramics, with a high pass filter at 1286 µm.

## 1. Introduction

Surface topographies, appropriately characterized, can explain a variety of physical phenomena, like wear and diffusion [1,2]. ISO 25178, EUR 15178N and ASME B46.1 standardize calculations of topographic and roughness characterization parameters. These parameters are classified according to their types: height, spatial, hybrid, function, or feature. Height parameters (*Sa, Sq, Sz, Ssk, Sku, Sp, Sv*) are the most used parameters. Nevertheless, in some cases, they are not sufficient, which is why hybrid or feature parameters, such as *Sdq* the root mean square gradient, *Spc* the arithmetic mean peak curvature, and the peak radius of curvature, are used. These parameters are related to the surface slopes and curvatures. They are calculated from the heights (*z*) and positions (*x, y*), and are averaged over the entire surface. Bataille et al. [3] studied the stick-slip phenomenon present on rods and showed that the average roughness (*Sa*) is not relevant while average slopes (*Sdq*) explain this phenomenon. Van Gorp et al. [4] demonstrated that the brightness of surfaces abraded by paper is directly correlated to the peaks’ radii of curvatures. Gloss correlates more strongly with the radius of curvature than it does the paper grade.

However, curvatures can better explain certain aspects of interface physics than topographies. Surface curvatures play important roles in diffusion [5], in aerodynamics [6], in friction [7], and in oxidation [8]. Curvature is suitable for the characterization of surfaces obtained by additive manufacturing [9]. Vulliez et al. [10] found strong correlations (*R²* > 0.95) between the surface curvatures and the fatigue limit of machined steels over a narrow range of scales, demonstrating that the scale of curvatures is important. Bartkowiak et al. [11] also found a strong correlation (*R²* > 0.85) between the coefficients of friction measured during bending under tension and the curvatures of milled and manually polished surfaces.

Essentially, curvatures are changes in slopes, and slopes are changes in height. When these first and second spatial derivatives are calculated from digitized 3D topographies, they are more sensitive to local amplitude variations than classic characterization parameters for roughness and form. Bartkowiak et al. [12] demonstrated that the conventional height parameters give information only at large scales, while multiscale curvature characterizations detect many topographical changes at all scales. These spatial derivatives can be calculated from different methods. In their article, Maleki et al. [13] compared five curvature characterization methods for fractal surfaces, sine waves, and real engineering surfaces, and proved that the Bigerelle–Nowicki [14] method best quantifies curvatures and differentiates surfaces. Nevertheless, derivatives amplify noise.

Rather than calculate curvatures from measured heights, an alternative is to use reflectance imaging directly to estimate the normal field on surfaces and, thus, local slopes. Reflectance transformation imaging (RTI) is frequently used to measure local spatial angular reflectance on surfaces. With RTI only light directions vary during data acquisitions. More on RTI implementation is explained in Section 2.2. In these reflectance measurements, variations in brightnesses of pixels with respect to illumination directions are used to calculate topographic gradients, i.e., slopes, or inclinations, of pixel-sized regions on surfaces [15]. This avoids discretization errors created during spatial differentiations of topographic height data to calculate slopes and curvatures, although one differentiation is still necessary to calculate curvatures from fields of normals. One advantage of RTI is that the measuring apparatus can be used in more hostile environments than conventional topographic measurement instruments, which are most often designed for laboratory use. Issues that need to be addressed include scales for characterizations and analyses, as well as appropriate statistics for topographic heterogeneities [2].

The objective of this paper is to demonstrate the suitability of surface gradient characterization by light reflectance (SGCLR) to study topographies of metal–ceramic composites worn in industrial conditions. The approach for this study includes: characterization of reflectance data by curvature tensors; segmenting composite surfaces by color; using curvatures in place of heights to characterize surfaces; and testing correlations of topographies with toughness by indentation tests. Conventional 3D characterization parameters (ISO 25178, EUR 15178N, and ASME B46.1) are calculated on curvatures instead of traditional heights. Multiscale analysis using a progression of different band pass filters is performed on curvature maps to find relevant scales for discriminating and correlating these surface topographies [2]. Multiscale light scattering by topographies, modeled as reflective facets, was simulated by Shipulski et al. [16]. This is studied experimentally by SGCLR, as well as wear characterization. SGCLR can be used as a quality control method whether it is the inspection of surfaces made by additive manufacturing processes [17], or the aesthetic aspect of fabrics [18] or jewelry.

## 2. Materials and Methods

### 2.1. Materials

#### 2.1.1. Selection of Surfaces

Table 1 lists the characteristics of surfaces selected to test some of the abilities of SGCLR to characterize surfaces.

From these specifications, six metal matrix composite (MMC) surfaces were selected. They were cut by electric discharge machining (EDM) from used rock crusher inserts. All the surfaces were subjected to identical loading conditions over the same time (C1, C2). 

Abrasion is the main wear mechanism in crushers (C3). Indeed, crushed raw materials are composed of abrasive particles, like quartz, that intensify the wear phenomenon. Systems are then sensitive to particle sizes [19] and concentrations of quartz present in crushed material [20]. This abrasion causes scratches and cracks on the grinding surface [21]. Some studies on different types of crushers were performed in order to base predictions on experiments [21,22] or numerical calculations [23,24] of wear mechanisms in crushers. Archard’s model [25] was adjusted to study this [22]. Pressure distributions and conditions, where wear rates are high, are discussed in the literature [21,22,26]. Pressure distributions depend on the raw material bed thickness and modifies the geometries of crushers. However, except for these predictive studies, this wear phenomenon has never been controlled and characterized by topographic analysis, perhaps due to difficulties measuring the surfaces. These surfaces are adequate for testing the abilities of SGCLR.

**Figure 1:**
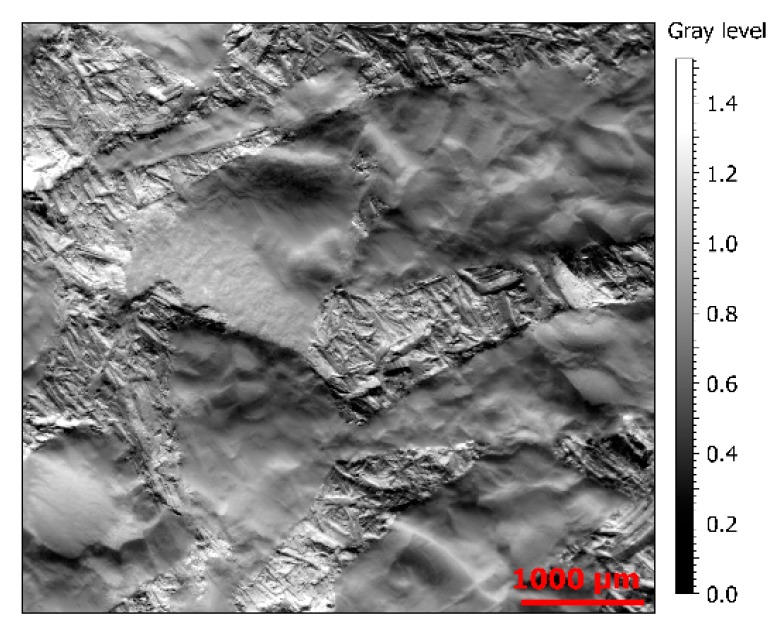
Rendering of Y-gradients (slopes) of a measurement on surface from material 3 (Section 2.2) (5140 × 4797 µm).

Figure 1 presents Y-gradients, i.e., slopes, on a portion of surface 3, on which some elementary mechanisms, such as abrasion scratches, can be observed. See Section 2.2 for explications about the gradient map.

**Figure 2:**
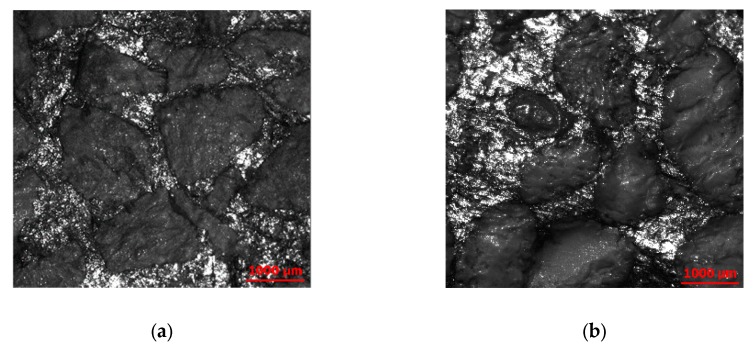
Images of (**a**) material 2 with black, cubic grains, and (**b**) material 5 with white, spherical grains. Both are 5140 × 4797 µm.

The materials are proprietary, metal–ceramic composites. They have a two-phase *α/β* structure. The *α*- and *β*- phases correspond to ceramic grains and metal alloy binders, respectively (C4). Each composite material is composed of the same metal, while differing in type and composition of the oxide ceramics (Figure 2). The ceramics have a high (50–80%, surfaces 1 and 2), a medium (30–50%, surfaces 5 and 6) or a low alumina content (10–30%, surfaces 3 and 4). There are two grain colors (C5), black (Figure 2a) and white (Figure 2b). The grain size is approximately 1500 µm for all ceramic grains. Figures of scanning electron microscope (SEM) are available in Appendix A.

Color is used to segment metal and ceramic portions of the surfaces with binary masks with MATLAB^®^ (MathWorks, Natick, MA, USA) and applied to images with Mountains Map^®^ (Digital Surf, Besançon, FR). High pass and low pass filters are combined to create band pass filters for multiscale analyses. These multiscale analyses characterize wear topographies at different scales and elucidate differences between materials, and between *α/β* structures. The former is an inter-comparison of one MMC versus another. The latter is an intra-comparison of ceramics versus metal.

#### 2.1.2. Mechanical Testing by Micro-Indentation

**Figure 3:**
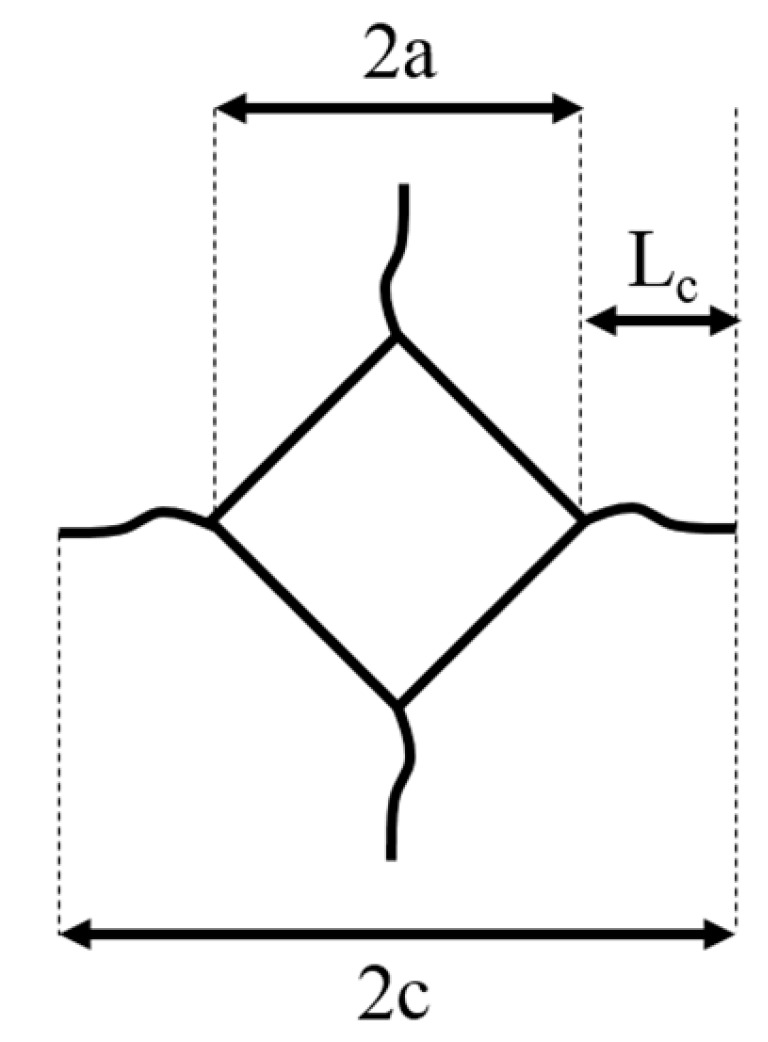
Diagonal and crack lengths of a Vickers indentation.

Vickers microhardness indentations were made with a pyramidal diamond indenter and a 5 kg load (DIN EN ISO 6507), once on five different grains of each ceramic. The hardness was determined from the indentation diagonal, 2*a* (Figure 3).

In 1976, Evans et al. [27] introduced Equation (1) to approximate of the fracture toughness *K_C_* from the length *a*, the length *c* and the Vickers hardness *H_v_*. The fracture toughness of each ceramic was determined by measuring *2a* and *c,* which includes *L_c_* the length of the crack generated by the indentation (Figure 3):

(1) $$K_{C}=0.16{\left(\frac{c}{a}\right)}^{-3/2}H_{v}a^{1/2}$$

Table 2 shows mechanical properties of six prepared surfaces. Values listed in the table are averages of five measurements on each surface.

### 2.2. Reflectance Measurements

#### 2.2.1. Principles

Reflectance is part of light interacting with surfaces. Reflectance data can be dense and complex. The most exhaustive description of reflectance, so far, has been obtained by BRDF (bidirectional reflectance distribution function) measurements. This information is estimated at a point, each location of each point is represented by a pixel. It is time intensive and includes large volumes of data. This can be too much for measuring on real, industrial surfaces, which are often textured and heterogeneous. Simplified measurement methods for reconstructing visual appearances of surfaces with image relighting were developed and are grouped under the name reflectance transformation imaging (RTI).

RTI is probably best known in cultural heritage communities, where it is used to study surface topographies of art and cultural heritage objects [28,29]. RTI is increasingly used in industry for inspecting surface defects on smoother surfaces [30,31,32], and, more generally, for providing appearances of industrial surfaces, for understanding how they are perceived visually [33]. 

**Figure 4:**
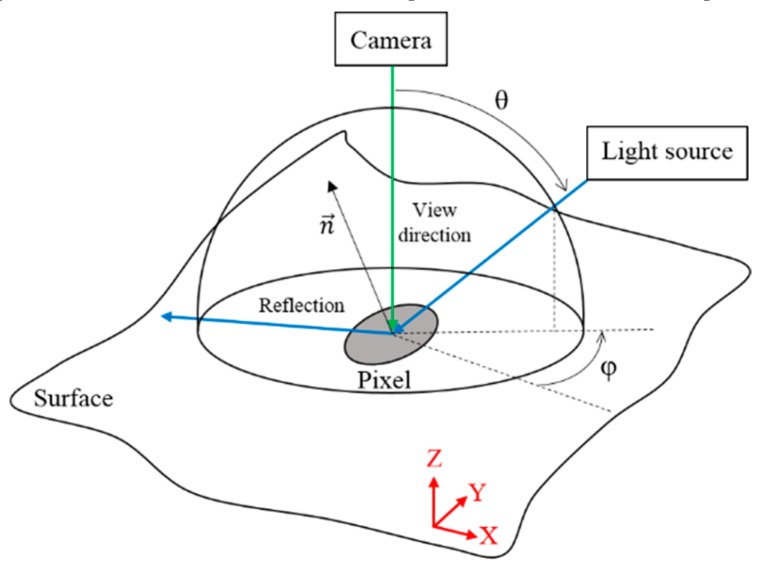
Reflectance transformation imaging.

Initially developed by Hewlett Packard labs as polynomial texture mappings ( PTM) [34], RTI measures only angular components of reflectance. Positions and directions of illumination are varied, while photographing the surface from a fixed position orthogonal to the surface to be measured. Images of the surface are captured, with different light directions (Figure 4).

Unit vectors normal to the surface are obtained from this stereo-photometric data, providing slopes. Curvatures are estimated from spatial derivatives of the slopes. Topographic maps can be estimated integrating the slopes. Here, the surface normal vectors are calculated from the captured images using mathematical models, such as, PTM [15], hemispherical harmonics (HSH) [35] and discrete modal decomposition (DMD) [30,36], or by photometric methods [37]. The latter is chosen in this study.

Indeed, Macdonald [38] demonstrated that surface normals computed from PTM coefficients are not as good as those obtained from photometric methods because of a smoothing approximation. HSH and DMD significantly improve appearance reconstructions for image relighting applications [30], allowing better approximations of angular variations of reflectance at each pixel. Descriptors from these models could improve the quality of the normal field estimation. However, no results related to this appear in the literature, therefore, in this study, the photometric method is used for estimation of normal vector fields, as detailed above.

Macdonald introduced the normals Equation (2) extracted from Woodham et al. [34]:

(2) $$\rho n=L^{-1}I$$

where *I* is the pixel intensity vector, *L* is the illumination positions matrix in cartesian coordinates, **n** are the normal vectors, and *ρ* is the maximum surface reflectance. *ρ* normalizes the normals. 

**Figure 5:**
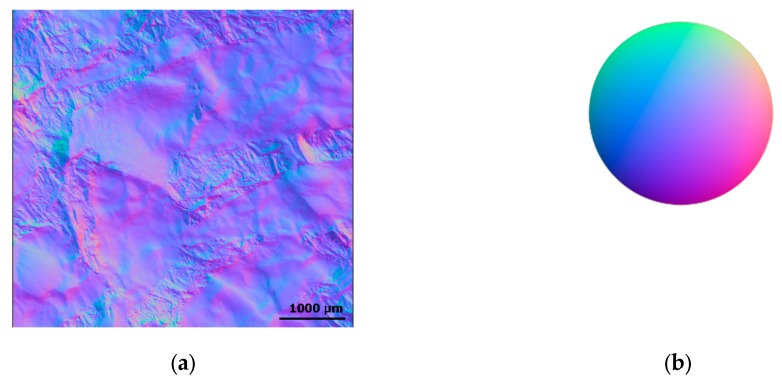
Rendering of a normal map (gradients or slopes) of a measurement of surface from material 3 (**a**). The direction of the vector is indicated by the color mix, where Red = X, Green = Y and Blue = Z (**b**).

Knowing illumination angles and corresponding pixel intensities, it is possible to determine local orientations, i.e., normals at each surface pixel. A representation of a normal field estimated on a metal–ceramic surface (material 4) is presented in Figure 5. Slope maps, also called inclinations or local gradients, can be estimated from the normals, by projecting normal vectors in the direction of the slopes. The curvature tensor is estimated by proceeding to a spatial derivative of the slopes. Curvatures, obtained by a single derivation operation, are expressed in two orthogonal directions. 

The eigenvalues (*K_1_*, *K_2_*), are the principal curvatures. These are the extreme values, maximum and minimum, of curvatures locally. Their orientations, the principal directions, are orthogonal. The principal curvatures and directions comprise the curvature tensor.

Once a curvature tensor is obtained, different curvatures descriptors can be calculated. These are generally based on the different curvature invariants. The most common are Gaussian curvatures *K_g_*, mean curvatures *H*, and Mehlum curvatures *K_Mehlum_* [39]:

(3) $$K_{g}=K_{1}\cdot K_{2}$$

(4) $$H=(K_{1}+K_{2})/2$$

(5) $$K_{Mehlum}=3/2\cdot H^{2}-1/2\cdot K_{g}$$

These invariants are intrinsic characteristics of the surface. They are independent of arbitrary choices of surface positions, or data, during measurement and calculation.

**Figure 6:**
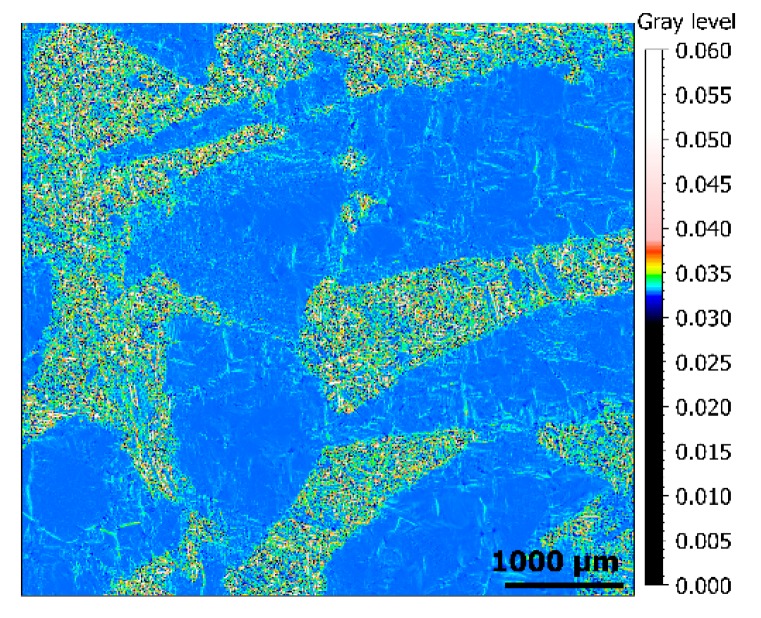
Rendering of a Mehlum curvature map of a measurement of surface from material 3.

These curvature descriptors are calculated at each pixel, so they can be expressed as a spatial function on the surface of the type *K_Mehlum_* = *g(x,y)*, creating a Mehlum curvature map (Figure 6). Instead of the usual heights (*z*), these functions, derived from gradients and calculated from reflectance measurements as described above, are used in this study to characterize the surface topographies.

#### 2.2.2. Topographic slope acquisition by reflectance transformation imaging (RTI)

**Figure 7:**
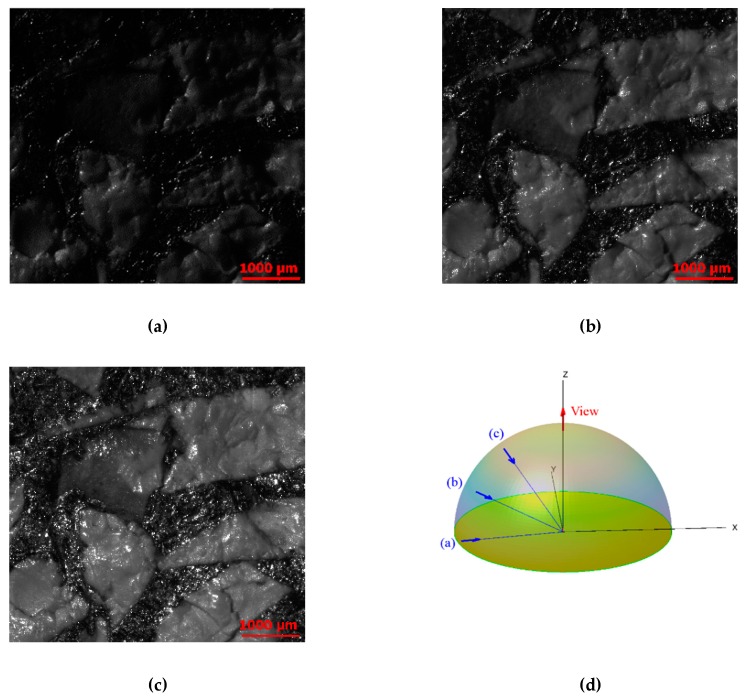
Reflectance images at ϕ = 215°. Θ = 80° (**a**), θ = 57.5° (**b**), θ = 35° (**c**), and reflectance halfsphere (**d**).

For the RTI acquisitions, a monochromatic camera was positioned above the surface to be measured. Illumination was provided by a white, LED, collimated high-quality, high-power light. Images associated with 360 individual angular positions of the light were acquired for each measurement. The positions included 72 *ϕ*-positions (azimuth), from 0° to 360°, by 5° increments, each with five *θ*-positions (elevation) from 35° to 80° (Figure 4 and Figure 7). Each image had a resolution of 1500 × 1400 pixels over a region of 5140 × 4797 µm, resulting in a pixel size about 3 × 3 µm.

### 2.3. Quantification of the SGCLR Relevance

**Figure 8:**
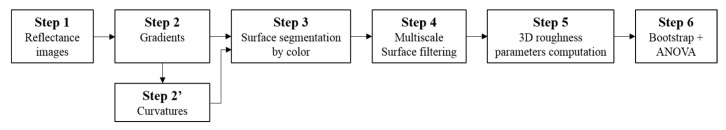
Data treatment.

The steps for data treatment and computation, as shown in Figure 8, are detailed in this section.

**Figure 9:**
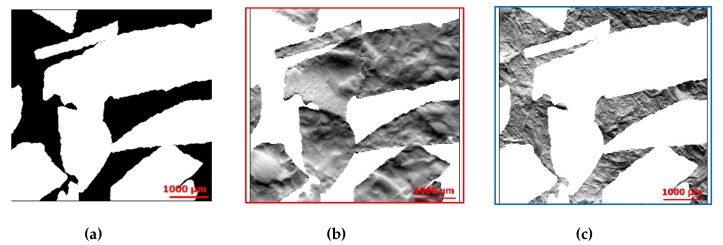
Segmentation of the surface 3, Y-gradient map. The binary mask was generated with a MATLAB^®^ algorithm (**a**), and ceramic (**b**) and metal (**c**) phases were obtained after application of the mask (MountainsMap^®^).

Step 1. Reflectance images, described in Section 2.2.2.
Step 2. Gradients and curvatures computation, described in Section 2.2.1.
Step 3. Surface segmentation: images were segmented, with an algorithm in MATLAB^®^, according to the colors of the ceramic and metal phases. The algorithm draws shapes by following edges of color regions. The generated binary mask (Figure 9a) was imported into MountainsMap^®^ and was applied to the shapes on each map (Figure 9b,c) in order to separate the ceramic grains from the metal phase.

Step 4. Filtering was done in MountainsMap^®^ for multiscale decompositions, with curvatures replacing heights. Gaussian filters were applied for low pass, high pass, and band pass, to all the ceramic and metal sections of the curvature maps, with 59 cutoff wavelengths *ε* varying from 2.2 to 4413 µm. High pass filtering keeps higher spatial frequencies, shorter spatial wavelengths, corresponding to roughness (low cutoffs). Low pass filtering keeps low spatial frequencies, longer wavelengths (high cutoffs), corresponding to waviness and form. Band pass is calculated by applying a high pass filter on the surface at a given cutoff *ε* and finally a low pass filter on the filtered surface at the cutoff *ε*-1.
Step 5. From these topographic representations of curvatures, within color segmentations, decomposed by multiscale filtering, 3D topographic characterization parameters were calculated in MountainsMap^®^. A total of 75 topographic characterization parameters were studied (ISO 25178, EUR 15178N, and software modules) treating curvatures as if they are heights.
Step 6. Statistical analyses by bootstrapping [40] and analysis of variance (ANOVA) [41] were done to determine the relevance (*F*) of different characterization parameters for discriminating the ceramics. A relevance index (*RI*) is calculated from the relevance *F*, the 95th percentile and the 5th percentile in order to normalize values:

(6) RI=F/(P95−P5)

The higher the relevance index, the more relevant the parameter associated to this index value is for discriminating surfaces.

## 3. Results and Discussion

**Figure 10:**
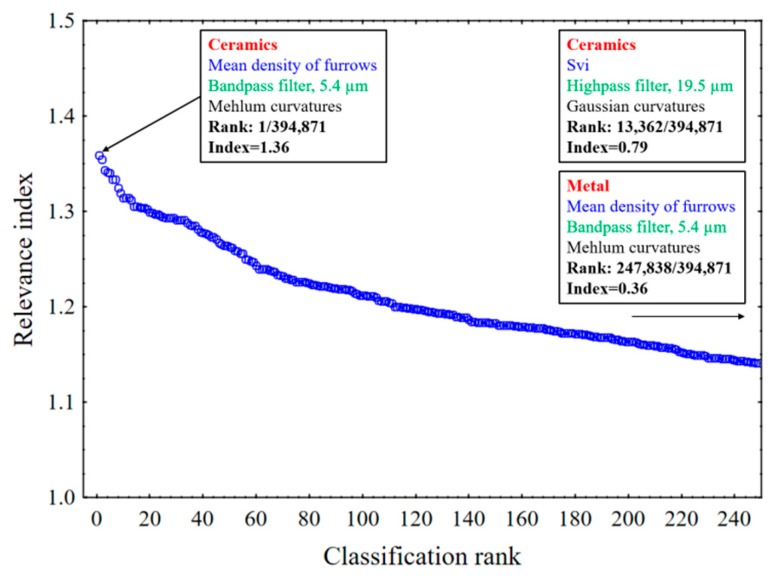
Classification rankings by relevance indices for discrimination of the seven materials by 75 topographic parameters for 100 bootstraps.

Figure 10 shows relevance indices versus classification orders, i.e., rankings, obtained for one hundred bootstraps. The mean density of furrows (MountainsMap^®^) is the most relevant parameter with a relevance index of 1.36 and a relevance, *F*, of 63. The mean density of furrows is statistically relevant for discriminating ceramic phases with Mehlum curvatures and a band pass filter at 5.4 µm.

**Figure 11:**
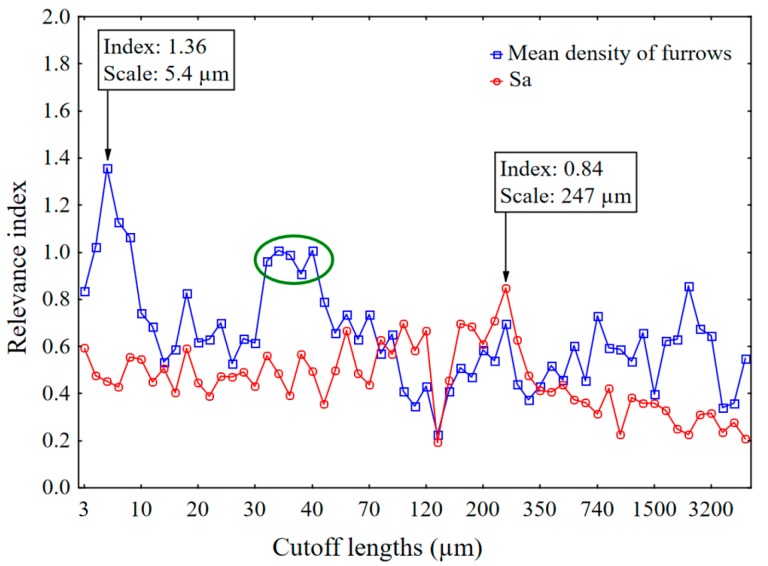
Relevance index versus cutoff lengths for the mean density of furrows and Sa. Ceramics, Mehlum curvatures and a band pass filtering. Classification rankings by relevance indices for discrimination of the 7 materials by 75 topographic parameters for 100 bootstraps.

Figure 11 compares relevance indices and cutoff lengths for mean densities of furrows and average roughness (*Sa*). *Sa* is not statistically relevant with a lower relevance index than the mean density of furrows.

**Figure 12:**
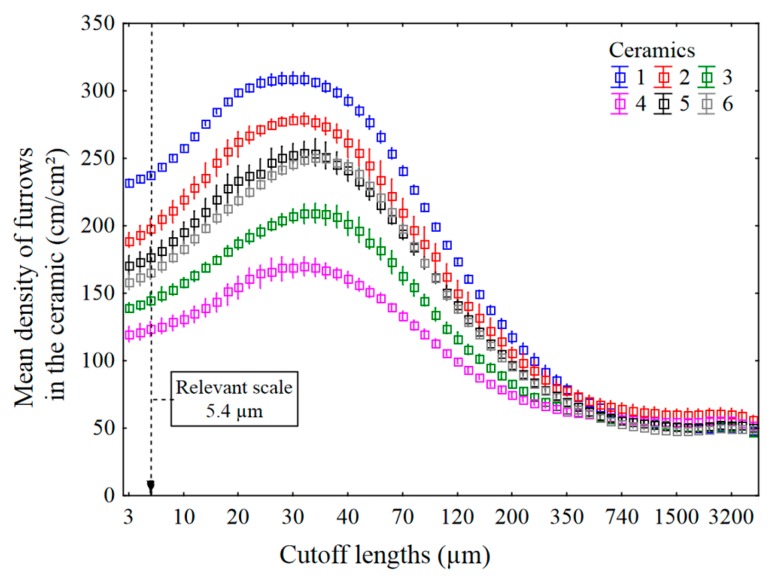
Mean density of furrows for all six ceramics versus multiscale cutoff lengths. Mehlum curvatures and a band pass filtering.

The mean densities of furrows are plotted versus cutoff lengths in Figure 12. The ceramics are best discriminated at a scale of 5.4 µm. Ceramic 1 has a larger density of furrows than ceramic 4. In topography, the density of furrows is the number of deep lines or scratches detected by patterns in curvature per unit area. In this study, a furrow represents a line of curvature sign changes of relatively close amplitude, at a particular scale.

The finest scale of the small plateau in relevance from 30 to 60 µm (Figure 11) corresponds to the scale of the maximum of the mean density of furrows for all ceramics (Figure 12). The mean density of furrows is different for all the ceramics; however, the scale of the maximum is about the same. All the curves in Figure 12 follow the same tendency, suggesting that this results from similar wear mechanisms during rock crushing.

**Figure 13:**
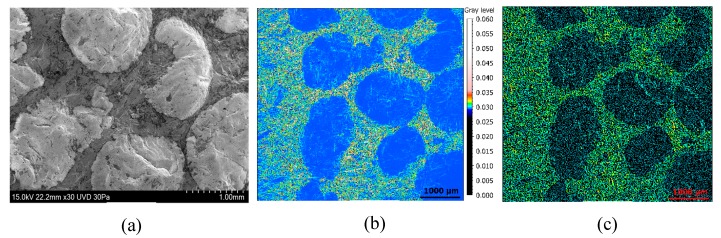
SEM image (**a**), Mehlum curvature map (**b**), and furrows map (**c**) of a measurement of the surface 4.

The Mehlum curvature maps calculated from reflectance acquisition are compared to SEM figures in Figure 13 and Appendix A. The SEM images are obtained with a Hitachi SU5000 at low vacuum. Ceramic grains are visible with difficulty contrary to the SGCLR method which allows to correctly isolate grains from metal phase (Figure 13a,b). Moreover, a SEM investigation shows that it is difficult to see and characterize the damage structures, no damage difference is distinct. The SGCLR method is then more suitable for characterizing grains damage and more precisely, with the Mehlum curvature which best discriminates ceramics (Figure 13b).

**Figure 14:**
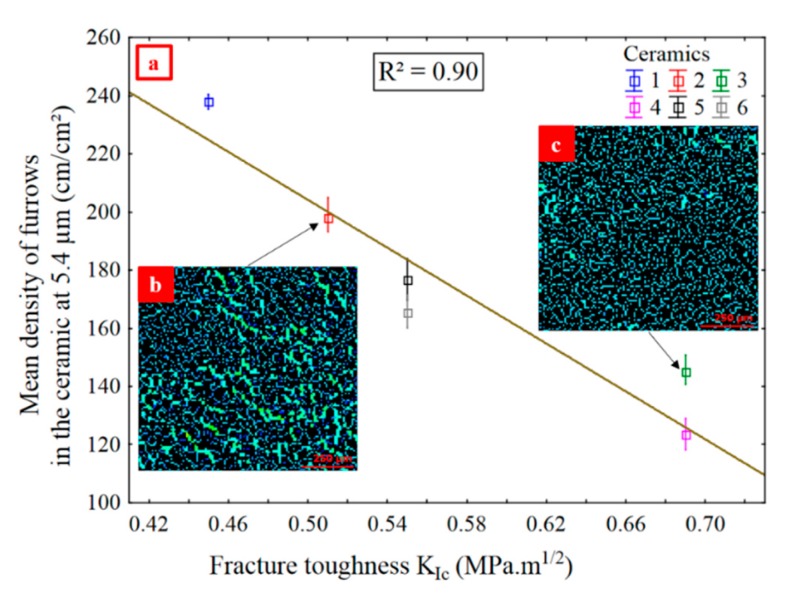
Mean density of furrows versus K_Ic_, for ceramics, Mehlum curvatures and a band pass filtering at 5.4 µm (**a**). Density of furrows in grains of ceramic 2 (**b**) and ceramic 3 (**c**), calculated from acquisition images at the same light angular position. MountainsMap^®^ module.

The density of furrows is compared to mechanical properties at the relevant scale of 5.4 µm for Mehlum curvatures and for a band pass (Figure 10 and Figure 12). A strong correlation is found between the density of furrows and fracture toughness *K_Ic_* (*R²* = 0.90) (Figure 14a). A regression analysis, based on a single bootstrap of 100 iterations, seeking the best correlation between the 3D parameters and the mechanical properties confirmed that the density of furrows for the ceramics with the Mehlum curvatures and a band pass at 5.4 µm is one of the parameters with the strongest correlation. The valley fluid retention index (*Svi*) for the ceramics with the Gaussian curvatures and a low pass at 19.5 µm has the strongest correlation with an *R²* value of 0.99, but *Svi* is not a good parameter for discrimination (Figure 10). 

The less the ceramic is resistant, the longer the crack length *L_c_*, the lower the *K_Ic_*, and the more the ceramic surface suffers damage in use, and the density of furrows is greater.

Akono et al. [42] carried out a study to determine the fracture toughness of a material from scratch tests. As demonstrated in their article, a correlation exists between the scratch parameters, width (*w*) and depth (*d*), and the vertical (*F_V_*) and horizontal (*F_T_*) forces applied during tests (Equation (7)):

(7) $$\sqrt{\left(1/2\cdot {F_{T}}^{2}+3/10\cdot {F_{V}}^{2}\right)}=K_{Ic}w\sqrt{d}$$

They showed that *K_Ic_* obtained from scratch testing is close to that measured by conventional mechanical testing. They performed tests on brittle materials, such as, cement paste and sandstone, for different scratch widths and depths of cut. For both materials, *K_Ic_* decreases with an increase of width which means that damage scratches are wider for a less resistant material at a given scratch depth. This is consistant with Figure 14b,c showing the furrows of more and less damaged ceramics, 2 and 3, respectively. Ceramic 2 is less crack resistant than 3, and has more pronounced furrows. The more a brittle material is crack resistant, the more it resists abrasion scratches [43].

The Mehlum curvature could be described as a minimization of elastic energy of a shape [44]. The Mehlum curvature is notably used in CAD to optimize the form of a system [44]. The furrows, represented by canyons, then correspond to paths of minimization of elastic energy. This is in agreement with the theory that the damage path corresponds to the path of low energy [45,46]. Ceramics with a high *K_Ic_* and low alumina content have less furrows because they are more elastic. Moreover, SEM investigations are made at the relevant scale of 5.4 µm to justify the statistical result. Micrometer-sized scratches are observed locally which confirms the scale of 5.4 µm (Appendix B). The scratches damage less the microstructure of the ceramic grains with the highest *K_Ic_* (Appendix B). However, these are local observations (10 x 10 µm) and not observations at the grain scale (1000 × 1000 µm). These do not represent the damage of the entire grain but justify locally the furrow phenomenom. The SGCLR method then characterizes better the damage because it averages on all grains and not a particular area of a grain.

**Figure 15:**
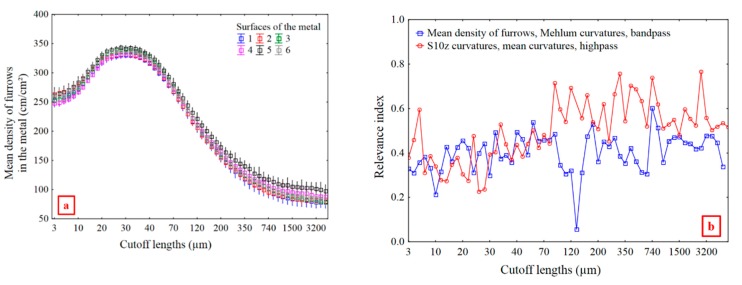
Mean density of furrows vs multiscale cutoff lengths for metal, Mehlum curvatures, and a band pass filtering (**a**). Relevance index versus cutoff lengths for the mean density of furrows for Mehlum curvatures and a band pass (blue), and S10z curvatures for mean curvatures and a high pass (red) (**b**).

There appears to be little difference in the mean density of furrows in the metal surfaces when used with different ceramics, as shown in Figure 15a. This does not appear to be relevant for discriminating surfaces of the metal, especially because of its low relevance index of 0.36 (Figure 10). 

*S10z* of mean curvatures (*H*, Equation (5)) with high pass filtering can be a relevant parameter (Figure 15b in red). For mean curvatures calculated from reflectance acquisitions, *S10z* is the difference between the five highest and the five lowest local curvatures defined in each motif of the Wolf Pruning decomposition. These are curvature variations between regions on entire metal surfaces. Consequently, *S10z* of mean curvatures can be related to damage heterogeneities.

**Figure 16:**
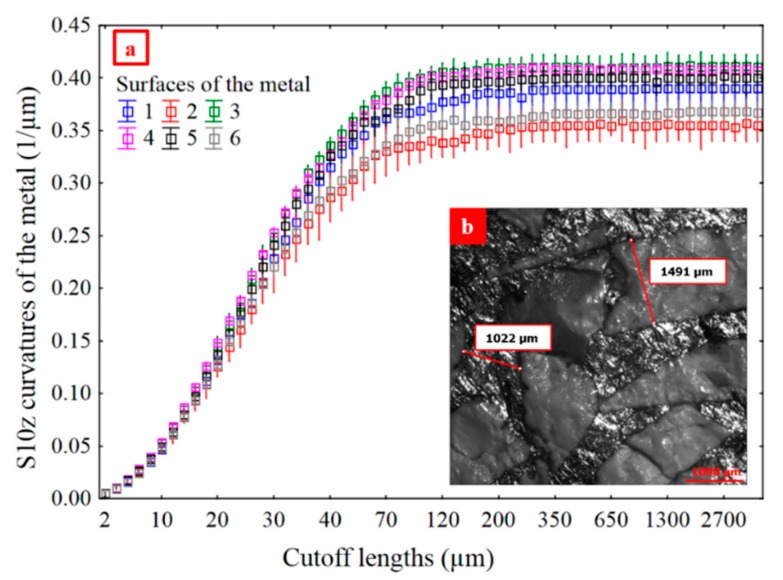
S10z vs. multiscale cutoff lengths for metal, mean curvatures and a high pass filtering (**a**). Common scale of the ceramic and the metal phases (**b**).

Distinct fluctuation regions of relevance indices for *S10z* of curvatures are evident in Figure 15b. One, between 3 and 70 µm, has relevance indices around 0.4. Another, between 80 and 2300 µm, has relevance indices around 0.65. At higher scales, the relevance indices show little fluctuation. In Figure 16a, the six *S10z* curves appear to be asymptotic to their maxima from 350 to 4000 µm. The “mean” scale of the asymptotic region, 1300 µm, is used to filter waviness for the ceramic and metal surfaces. The ceramic grain size and spacing, which corresponds to sizes of the metal phases, is in the order of 1000 µm (Figure 16b). The scale of relevance corresponds to intra-metal and intra-ceramic curvature variations.

**Figure 17:**
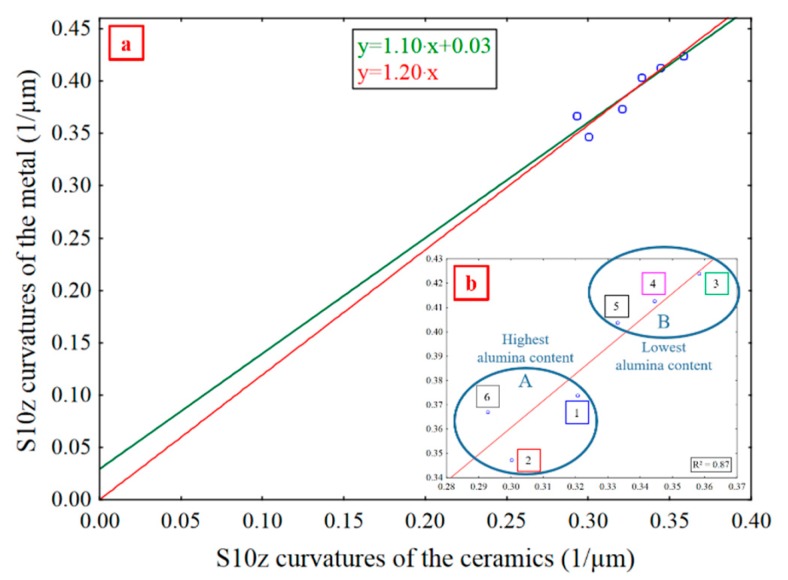
*S10z* curvatures of the metal vs *S10z* curvatures of the ceramics. Affine and linear approximations (**a**), and linear model (**b**). Ceramics and metal, mean curvatures, and a high pass filtering at 1286 µm.

Figure 17 shows *S10z* for metal curvatures versus *S10z* for ceramic curvatures with a high pass cutoff filter at 1286 µm. There is a linear relation, y=ax+b where, a=1.2 (*p* = 7 × 10^–6^) and b=0.03 (*p* = 0.50) (Figure 17). Because *b* is small, this can be approximated as $S10z_{metal}=1.2\cdot S10z_{ceramic}$ (*p* = 0, *R²* = 0.87). With the intercept at the origin, the damage is always similar in the ceramics and the metal, providing a statistically robust model for comparing damage in the ceramics and the metal. This indicates that ceramic and metal damage are co-dependent [43]. The more the ceramic is plane, i.e., with a curvature close to zero, then undamaged, the more the metal is too. The metal damage variation increases with the ceramics’ damage. As the metal is more ductile than the ceramic, its efficiency of abrasion is low [47].

Since the ratio of *S10z* curvatures of the metal to that of the ceramics, with a high pass filter at 1286 µm, is 1.2, the *S10z* curvatures of the metal is 20% higher than that of the ceramics, indicating that the metal suffers 20% more damages than the ceramics. Curvature variations on the metal surface are more heterogeneous because of its ductility, for a given tribosystem, while the ceramics are more stable and resistant. The ceramics have less complex damage variations than the metal.

There are two distinct damage groups (Figure 17b). Group A (1, 2, 6) contains the ceramics having the lowest fracture toughness with a mean *K_Ic_* of 0.51 and the highest alumina content (50–80%). Group B (3, 4, 5) contains the ceramics with the lowest alumina content (10–30%), the highest fracture toughness and a mean *K_Ic_* of 0.64. As scratch width decreases when fracture toughness increases at a given scratch depth [38], curvatures increase. That is why group B has higher *S10z* curvatures values, with a high pass filter at 1286 µm, than group A.

## 4. Conclusions

This study is the first to present multiscale topographical analyses of curvatures calculated by Surface Gradient Characterization of Light Reflectance. It is applied to several different worn MMC surfaces. Gradients and curvatures calculated from RTI (reflectance transformation imaging) acquisitions are mapped onto 3D surfaces and segmented to separate ceramic and metal phases. Conventional analysis methods, defined in ISO 25178, EUR 15178N, and ASME B46.1, intended for characterizing heights, are applied to gradients and curvatures. Consequently, new curvature-based characterization parameters are defined, with the mathematical expressions intended for heights. These are calculated in software intended for heights. Multiscale and statistical analyses can be performed on these curvature maps to investigate sensitivities to scales, and to determine the relevance of these curvature characterizations for discriminating different surfaces, damaged ceramics, and metal phases of MMC, independently.

1. The density of furrows for Mehlum curvatures and *S10z* for mean curvatures, curvatures calculated from reflectance acquisitions, quantify the wear of the ceramics and of the metal at different scales: small, i.e., high spatial frequencies for density of furrows for Mehlum curvatures, and large, i.e., low spatial frequencies for *S10z* for mean curvatures.
2. The density of furrows for Mehlum curvatures, at a scale of 5.4 µm, is the most relevant parameter for evaluating the wear difference between the ceramics.
3. The density of furrows for Mehlum curvatures, at 5.4 µm, is proportional to the number of scratches, which are indications of an elementary wear mechanism namely abrasive wear.
4. Material damage is related to mechanical properties. A strong correlation exists between the density of furrows for Mehlum curvatures, at 5.4 µm, and the fracture toughness (*R²* = 0.90). A material with a high *K_Ic_* presents less scratches.
5. A strong correlation (*R²* = 0.87) is found between the *S10z* for curvatures, with a high pass filter at 1286 µm, of the metal and the ceramics, with the metal more damaged than the ceramics.
6. There are no heterogeneities in the results showing any influence of the material color on the SGCLR. The SGCLR method is not sensitive to surface colors.

## Figures and Tables

**Table 1:** Specifications for testing SGCLR.

Criteria	Surface Characteristics	Can SGCLR…
C1	Different levels loading for the same wear mechanism	Quantify graduated morphological differences ?
C2	Multiscale topographical structure (fractal)	Detect different topographical scales ?
C3	Different wear mechanisms, abrasion versus spalling	Detect and quantify elementary physical mechanisms that are sources of gradients ?
C4	Composite with two materials with different topographies (metal matrix and ceramic)	Segment surfaces for determining morphological indicators discriminating zones ?
C5	Different surface colors	Be invariant with respect to colors ?

**Table 2:** Mechanical properties, and indentation and crack dimensions.

Surfaces	2*a* (µm)	*c* (µm)	*H_v_* (MPa)	*K_Ic_* (MPa√m)
1	78.9	88.6	1489	0.4565
2	77.8	83.2	1532	0.5029
3	82.0	67.5	1378	0.6817
4	84.9	68.2	1288	0.6829
5	84.1	76.9	1310	0.5526
6	82.7	76.5	1356	0.5566

## Nomenclature

*Abbreviations*						
ANOVA		ANalysis Of VAriance				
MMC		Metal matrix composite				
RTI			Reflectance transformation imaging			
SGCLR		Surface gradient characterization by light reflectance				
*Roughness parameters*						
*S10z*			Ten point height			
*Sa*			Arithmetic mean height			
*Svi*			Valley fluid retention index			
*Mechanical parameters*						
*α*			Ceramic phase			
*β*			Metal phase			
*d*			Depth of scratch			
*H_v_*			Hardness			
*K_Ic_*			Fracture toughness			
*L_c_*			Crack length			
*w*			Width of scratch			
*RTI and curvature parameters*						
*ϕ*			Azimuth			
*θ*			Elevation			
*H*			Mean curvature			
*K_1_*			Minimum principal curvature			
*K_2_*			Maximum principal curvature			
*K_g_*			Gaussian curvature			
*K_Mehlum_*		Mehlum curvature				
*Multiscale and statistical parameters*						
*ε*			Cutoff length			
*F*			Relevance			

## Appendix A

	**SEM Images**	**Mehlum Curvature Maps**	**Furrows Maps**
**Surface 1** *K_Ic_* = 0.457 MPa√m 50–80% of alumina Black and cubic grains			
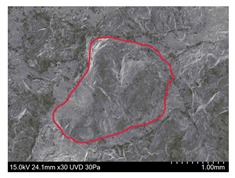
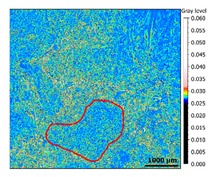
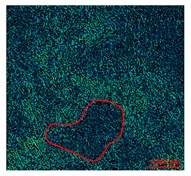
**Surface 2** *K_Ic_* = 0.503 MPa√m 50–80% of alumina Black and cubic grains			
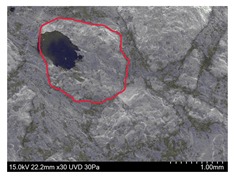
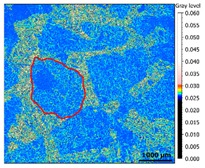
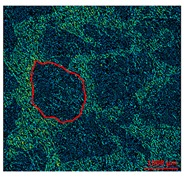
**Surface 3** *K_Ic_* = 0.682 MPa√m 10–30% of alumina White and cubic grains			
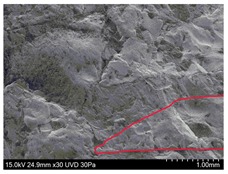
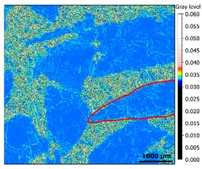
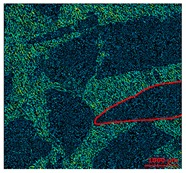
**Surface 4** *K_Ic_* = 0.683 MPa√m 10–30% of alumina White and spherical grains			
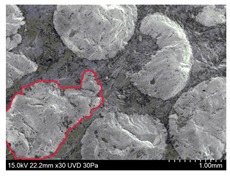
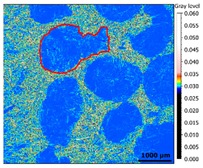
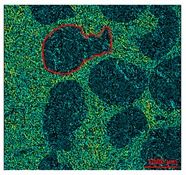
**Surface 5** *K_Ic_* = 0.553 MPa√m 30–50% of alumina White and spherical grains			
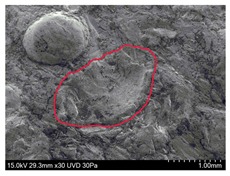
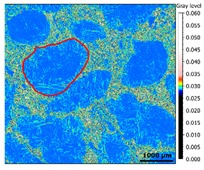
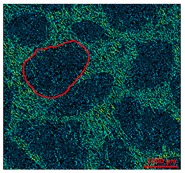
**Surface 6** *K_Ic_* = 0.557 MPa√m 30–50% of alumina White and cubic grains			
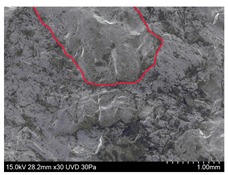
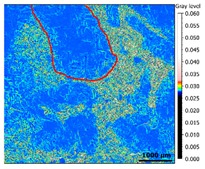
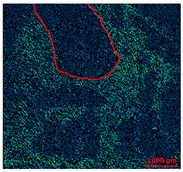

## Appendix B

**Surfaces**	**1 and 2**	**5 and 6**	**3 and 4**
**Alumina content**	High (50–80%)	Medium (30–50%)	Low (10–30%)
**Mean *K_Ic_*** (MPa√m)	0.48	0.55	0.68
**SEM zoom**			
